# Angioedema Secondary to IV Tissue Plasminogen Activator Administration for Treatment of Acute Ischemic Stroke

**DOI:** 10.1155/2018/3257215

**Published:** 2018-04-15

**Authors:** Benjamin Chaucer, Dustin Whelan, Christopher Veys, Manas Upadhyaya

**Affiliations:** ^1^University of Illinois College of Medicine at Peoria, 1 Illini Dr, Peoria, IL 61605, USA; ^2^Illinois Neurological Institute, OSF Saint Francis Medical Center, Peoria, IL, USA

## Abstract

**Background:**

IV tissue plasminogen activator (tPA) is the treatment of choice for ischemic strokes that present within the treatment window. In the majority of patients, this offers an effective and often life-prolonging treatment in the acute setting. In a rare set of patients treated with IV tPA, side effects can be seen. One rare and potentially dangerous side effect is angioedema.

**Case Report:**

We present the case of a patient treated for ischemic stroke who developed angioedema and discuss the etiology and risk factors for this rare, but dangerous side effect.

**Conclusion:**

Given the frequent and widespread use of tPA, awareness of the rare life-threatening side effects is paramount. This is of particular importance for practitioners in the acute care setting.

## 1. Introduction

Approximately 795,000 Americans experience a stroke each year with 87% of these being ischemic [1]. In 1996, IV tPA was approved for treatment of acute ischemic stroke and since then has been the standard of treatment for patients experiencing ischemic stroke [2]. Common side effects of IV tPA include intracerebral hemorrhage, systemic hemorrhage, and rarely angioedema. The incidence of angioedema increases in patients who also take an ACE inhibitor. We present the case of a 62-year-old female who presented with signs and symptoms of acute stroke and was treated with IV tPA that resulted in self-limiting angioedema.

## 2. Case Report

**Figure 1:**
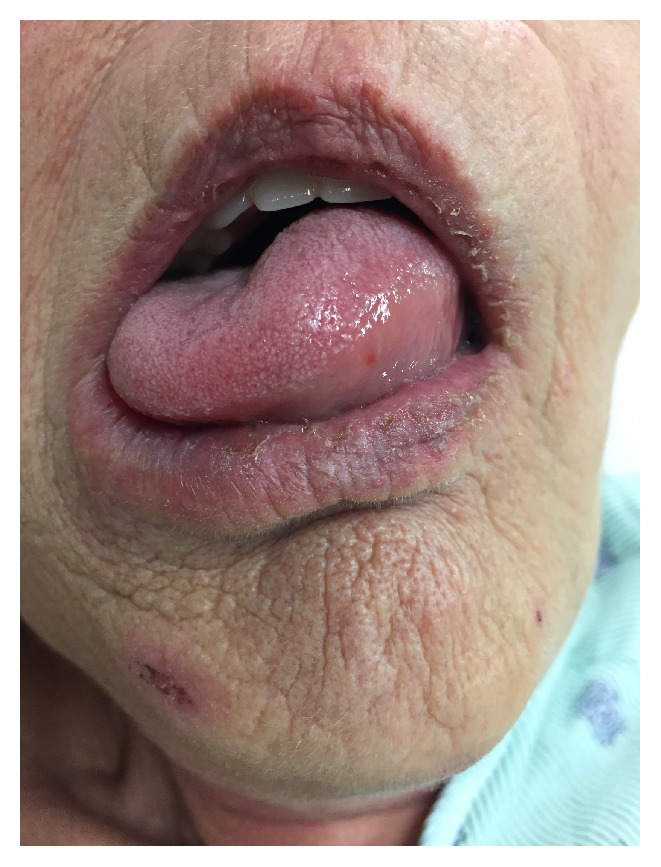
Image showing left lingual angioedema.

Patient is a 62-year-old female who presented to the emergency department as a stroke call due to symptoms of left sided weakness, facial droop, and slurred speech. Upon arrival by EMS, the patient was brought directly to CT for imaging where the neurology stroke team was awaiting to assess the patient. On presentation, patient's glucose was 123. Review of patients prior to admission medication showed that patient was taking Lisinopril 40 mg QD. On physical exam, patient had left sided facial droop with resulting slurred speech. NIHSS assessment was performed and found to be 6: 2 points for partial facial paralysis, 1 point for left arm drift, 2 points for sensory loss in left face, arm, and leg, and 1 point for mild slurred speech. CT head showed a tapered occlusion of the superior division of the right M2 segment, approximately 1.4 cm distal to the origin with resultant ischemic stroke of right M2 segment of the MCA. Patient was given IV tPA calculated from weight with a bolus dose of 7 mg and an infusion dose of 62.85 mg. Within 20 minutes of onset of tPA administration, patient started to complain of facial swelling. Reassessment revealed acute onset of swelling to the patient's left side of her tongue contralateral to the location of the stroke (see Figure 1). Inspection of the oral cavity revealed no trauma to the tongue or hematoma. Patient denied SOB or difficulty breathing and had SaO2 92%. A diagnosis of oral lingual angioedema secondary to tPA administration was made. Given lack of concerns for airway obstruction, patient was closely observed in the emergency room setting. Patient was found to have patent airway throughout the episode, so decision was made to hold treatment and monitor closely. Patient was stabilized and admitted to neurology intensive care unit for close observation after tPA administration and for initiation of stroke risk factor reducing therapies.

## 3. Discussion

Angioedema secondary to ACE inhibitor treatment is well documented with an incidence of 0.1%–2% [3]. Treatment of angioedema is driven by the inciting cause. Mild cases may respond to antihistamines, while more severe cases may require corticosteroids, epinephrine, and even bradykinin antagonists like Icatibant. Angioedema secondary to tPA administration is a rare side effect with an estimated incidence of 0.02% in patients being treated with alteplase for acute MI [4]. Several studies have shown that the incidence of oral lingual angioedema with tPA administration for stroke may be higher than previously understood. Angioedema secondary to tPA administration for acute stroke is estimated at 0.2–5.1% [5]. The documented incidence of angioedema secondary to tPA administration increases with the use of ACE inhibitor. Hill et al. described a 5-year study involving 176 patients treated with IV alteplase for acute stroke. In this study, 5.1% were found to have angioedema secondary to IV alteplase administration [6]. In this same study by Hill et al., 7 out of the 9 patients were found to have angioedema on the contralateral side of the ischemic stroke [6] as was seen in our patient. Given that angioedema was noted contralateral to the location of our patients stroke we postulate that this is likely due to ischemic changes. In our patient, the right M2 segment of the MCA was affected which may have resulted in autonomic dysfunction of the insular cortex. This may have been responsible for the resulting angioedema contralateral to the inciting stroke. In a previous study by Hill et al. of 105 patients given IV tPA, 2 were found to have subsequent angioedema, both of which were contralateral to the stroke [7]. However, the largest study performed by Hill and Buchan enrolled 1,135 patients and found an incidence of angioedema secondary to IV tPA administration of 1.3% [8]. This variation in resultant angioedema suggests that a clear causative etiology has not yet been established. Angioedema is the result of the unregulated release of histamine, bradykinin, and the compliment cascade. The mucosal tissue is commonly effected although dermal or subcutaneous tissues can be effected as well. Alteplase results in a secondary increase in bradykinin due to the cleavage of high molecular weight kininogen. This overproduction of bradykinin may in part be responsible for tPA induced angioedema. In patients like ours, who are currently taking an ACE inhibitor, this may compound the production of bradykinin. ACE inhibitors work by prohibiting the conversion of angiotensin I to angiotensin II. A secondary effect of ACE inhibitors is the decreased breakdown of bradykinin by inhibition of plasma kinases. This may in part explain the increased frequency of angioedema secondary to tPA administration in patient who are currently taking an ACEI. In this subset of patients, the use of an ACEI in tandem with tPA may act to increase bradykinin production while decreasing bradykinin destruction. This may in part explain the resulting angioedema. Case reports of angioedema are scarce and may be due to underreporting or lack of detection in the acute phase of tPA administration. Although usually self-limiting some cases have been documented of patients requiring intubation due to progressive angioedema [9]. Overlapping symptomatology may be responsible for the rarity of documented cases of angioedema secondary to IV alteplase. Although rare, given the possible gravity of angioedema, this case highlights an important adverse drug effect for practitioners who frequently use IV tPA.
